# Binding characteristics of [^18^F]PI-2620 distinguish the clinically predicted tau isoform in different tauopathies by PET

**DOI:** 10.1177/0271678X211018904

**Published:** 2021-05-27

**Authors:** Mengmeng Song, Leonie Beyer, Lena Kaiser, Henryk Barthel, Thilo van Eimeren, Ken Marek, Alexander Nitschmann, Maximilian Scheifele, Carla Palleis, Gesine Respondek, Maike Kern, Gloria Biechele, Jochen Hammes, Gèrard Bischof, Michael Barbe, Özgür Onur, Frank Jessen, Dorothee Saur, Matthias L Schroeter, Jost-Julian Rumpf, Michael Rullmann, Andreas Schildan, Marianne Patt, Bernd Neumaier, Olivier Barret, Jennifer Madonia, David S Russell, Andrew W Stephens, Andre Mueller, Sigrun Roeber, Jochen Herms, Kai Bötzel, Adrian Danek, Johannes Levin, Joseph Classen, Günter U Höglinger, Peter Bartenstein, Victor Villemagne, Alexander Drzezga, John Seibyl, Osama Sabri, Guido Boening, Sibylle Ziegler, Matthias Brendel

**Affiliations:** 1 Department of Nuclear Medicine, University Hospital of Munich, LMU Munich, Munich, Germany; 2 Department of Nuclear Medicine, University of Leipzig, Leipzig, Germany; 3 Cognitive Neuroscience, Institute for Neuroscience and Medicine (INM-3), Research Centre Juelich, Juelich, Germany; 4 Department of Nuclear Medicine, University Hospital Cologne, Cologne, Germany; 5 Department of Neurology, University Hospital Cologne, Cologne, Germany; 6 German Center for Neurodegenerative Diseases (DZNE), Bonn, Germany; 7 InviCRO, LLC, Boston, MA, USA; 8 Molecular Neuroimaging, A Division of inviCRO, New Haven, CT, USA; 9 Department of Neurology, University Hospital of Munich, LMU Munich, Munich, Germany; 10 Department of Neurology, Medizinische Hochschule Hannover, Hannover, Germany; 11 Department of Psychiatry, University Hospital Cologne, Cologne, Germany; 12 Center for Memory Disorders, University Hospital Cologne, Cologne, Germany; 13 Department of Neurology, University of Leipzig, Leipzig, Germany; 14 Clinic for Cognitive Neurology, University of Leipzig, Leipzig, Germany; 15 LIFE – Leipzig Research Center for Civilization Diseases, University of Leipzig, Leipzig, Germany; 16 Max- Planck-Institute of Human Cognitive and Brain Sciences, Leipzig, Germany; 17 FTLD Consortium Germany, Ulm, Germany; 18 Laboratoire des Maladies Neurodégénératives, Université Paris-Saclay, CEA, CNRS, MIRCen, Fontenay-aux-Roses, France; 19 Life Molecular Imaging GmbH, Berlin, Germany; 20 Center for Neuropathology and Prion Research, University Hospital of Munich, LMU Munich, Munich, Germany; 21 German Center for Neurodegenerative Diseases (DZNE), Munich, Germany; 22 Munich Cluster for Systems Neurology (SyNergy), Munich, Germany; 23 Department of Neurology, Technical University Munich, Munich, Germany; 24 Department of Molecular Imaging & Therapy, Austin Health, Heidelberg, VIC, Australia; 25 The Florey Institute of Neuroscience and Mental Health, The University of Melbourne, Melbourne, VIC, Australia; 26 Department of Medicine, Austin Health, The University of Melbourne, Melbourne, VIC, Australia

**Keywords:** Tau, PI-2620, binding, affinity, kinetic modelling

## Abstract

The novel tau-PET tracer [^18^F]PI-2620 detects the 3/4-repeat-(R)-tauopathy Alzheimer’s disease (AD) and the 4R-tauopathies corticobasal syndrome (CBS) and progressive supranuclear palsy (PSP). We determined whether [^18^F]PI-2620 binding characteristics deriving from non-invasive reference tissue modelling differentiate 3/4R- and 4R-tauopathies. Ten patients with a 3/4R tauopathy (AD continuum) and 29 patients with a 4R tauopathy (CBS, PSP) were evaluated. [^18^F]PI-2620 PET scans were acquired 0-60 min p.i. and the distribution volume ratio (DVR) was calculated. [^18^F]PI-2620-positive clusters (DVR ≥ 2.5 SD vs. 11 healthy controls) were evaluated by non-invasive kinetic modelling. R1 (delivery), k2 & k2a (efflux), DVR, 30-60 min standardized-uptake-value-ratios (SUVR_30-60_) and the linear slope of post-perfusion phase SUVR (9-60 min p.i.) were compared between 3/4R- and 4R-tauopathies. Cortical clusters of 4R-tau cases indicated higher delivery (R1_SRTM_: 0.92 ± 0.21 vs. 0.83 ± 0.10, p = 0.0007), higher efflux (k2_SRTM_: 0.17/min ±0.21/min vs. 0.06/min ± 0.07/min, p < 0.0001), lower DVR (1.1 ± 0.1 vs. 1.4 ± 0.2, p < 0.0001), lower SUVR_30-60_ (1.3 ± 0.2 vs. 1.8 ± 0.3, p < 0.0001) and flatter slopes of the post-perfusion phase (slope_9-60_: 0.006/min ± 0.007/min vs. 0.016/min ± 0.008/min, p < 0.0001) when compared to 3/4R-tau cases. [^18^F]PI-2620 binding characteristics in cortical regions differentiate 3/4R- and 4R-tauopathies. Higher tracer clearance indicates less stable binding in 4R tauopathies when compared to 3/4R-tauopathies.

## Introduction

Accumulation of hyperphosphorylated microtubule-associated tau protein (MAPT, tau) in neurons and glia is a hallmark of a wide range of neurodegenerative diseases.^1^ Disorders associated with the accumulation of MAPT are thus termed tauopathies, which include Alzheimer’s disease (AD) as well as progressive supranuclear palsy (PSP), corticobasal degeneration (CBD), and Pick’s disease (PiD) among the non-AD tauopathies. Accumulation of tau protein is closely associated with neurodegeneration and cognitive impairment.^2,3^

The neuropathology of different tauopathies exhibits varying isoform composition of their filaments as well as distinct neuroanatomical distribution and relative amounts of tau inclusions.^4^ Six molecular isoforms of tau are generated by alternative pre-mRNA splicing of a single gene transcript and classified according to the number of repeats of the microtubule binding domains as 3-repeat (3R) and 4-repeat (4R) tau proteins.^5,6^ Equal amounts of 3R and 4R tau isoforms exist in the normal brain. In tauopathies, tau is abnormally hyperphosphorylated and accumulates intracellularly, forming tangles of paired helical filaments (PHF), twisted ribbons and/or straight filaments.

AD accounts for the majority of tauopathy cases and is marked by predominantly PHF of tau with an approximately equal 3R to 4R ratio in the microtubule-binding domain, mixed with straight filaments.^7–9^ In contrast, PSP and CBD tau aggregation forms straight filaments of tau, which are primarily composed of the 4R isoform.^5,7,10–13^

Positron emission tomography (PET) emerged for non-invasive detection of tau inclusions in the brain during the recent years.^14,15^ Tau-PET tracers allow imaging of the presence and spatial extent of brain tau deposition, facilitating characterization and quantification in humans. First generation tau-radiotracers have already provided topographic distribution and quantitative estimates of tau pathology in AD and non-AD tauopathies closely matching the known patterns in autopsy.^16–21^ However, relevant amounts of the signal of first generation tau-PET ligands prove to be non-specific^22,23^ which accelerated the development of next-generation tau PET ligands with reduced off-target binding.^14,24–27^

For tau-PET tracers, binding to both 3R and 4R tau isoforms and different tau-folds would allow the detection of various tauopathies.^28^ In vitro pharmacological studies and autoradiography of the novel second generation tau-PET tracer [^18^F]PI-2620 demonstrated its potential to bind to both 3R and 4R aggregated tau isoforms as well as to different tau aggregate folds in AD and non-AD tauopathies using AD and PSP brain sections as well as brain homogenates of PSP and PiD patients.^25^ First in human results suggest that [^18^F]PI-2620 is able to visualize the predominantly 3/4R-tauopathy AD^29,30^ and the mainly 4R-tauopathies corticobasal syndrome (CBS)^31^ and PSP^32^ by PET, but likely with a different magnitude of affinity among them (pIC50 8.5 ± 0.1 for AD brain tissue versus pIC50 7.7 ± 0.1 for PSP brain tissue).^25^ The majority of Aβ-negative cases with a clinical phenotype of CBS obtained the neuropathological diagnosis of a 4R-positive CBD in autopsy.^33^ The lower binding affinity of [^18^F]PI-2620 to 4R tau *in vitro* could cause faster clearance from the target when compared to 3/4R tau *in vivo*. Although 3/4R and 4R tauopathies have obvious topographical differences in the majority of cases, a regional overlap exists among them. For instance, patients with AD-CBS (predominant 3/4R) and 4R-CBS (predominant 4R) are characterized by involvement of the motor cortex and patients with typical AD (predominant 3/4R) and argyrophilic grain disease (predominant 4R) comprise tau accumulation in parietal-temporal cortices.^34^ Thus, detection of differences in [^18^F]PI-2620 binding characteristics among different tau isoforms would potentially facilitate a more reliable differential diagnosis.

The aim of this study was to determine whether, [^18^F]PI-2620-positive cortical and subcortical clusters have different binding characteristics as assessed by non-invasive reference tissue modelling. Finally, we performed a receiver operating curve analysis to determine if clinically diagnosed 3/4R and 4R tauopathies can be differentiated by the tracer kinetics and binding characteristics of [^18^F]PI-2620.

## Material and methods

### Cohort and study design

**Figure 1.**
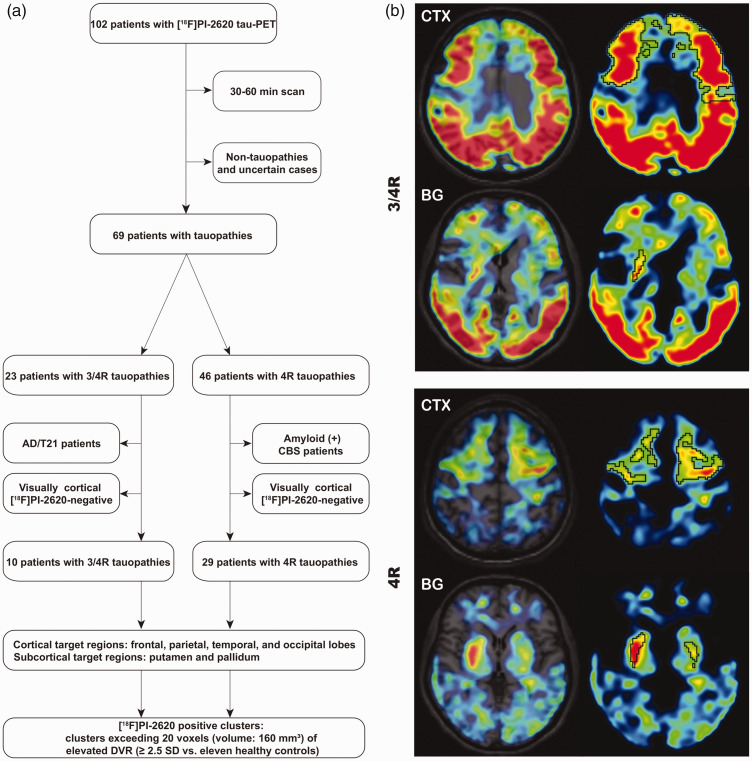
(a) Study flowchart. Only patients with a dynamic scan, clinical confidence on the diagnosis and a cortical [^18^F]PI-2620-positive PET scan were included. (b) Examples of cortical (frontal cortex) and subcortical target region definition by z-score maps in patients with clinically diagnosed 3/4 R (upper panel) and 4 R (lower panel) tauopathies. Clusters above a threshold of mean value + 2.5 standard deviations (black) were defined as [^18^F]PI-2620-positive assuming tau-positivity. CTX: cortex; BG: basal ganglia; AD: Alzheimer’s disease; CBS: corticobasal syndrome.

The subjects were either recruited and scanned at the Ludwig-Maximilians-University of Munich (LMU), Department of Nuclear Medicine or took part at the first in human study of [^18^F]PI-2620 at Invicro (New Haven, U.S.A.) between December 2016 and September 2020. Patients were diagnosed to belong to the AD continuum (total n = 10: mild cognitive impairment (MCI), n = 5; dementia due to AD, n = 5) as 3/4R tauopathy or PSP (n = 15)/CBS (n = 14) as 4R tauopathies according to current diagnostic criteria.^35–38^ In particular, the AD continuum patients were required to meet criteria for typical AD with MCI or dementia according to the diagnostic criteria of the National Institute on Aging and Alzheimer’s Association.^39^ Diagnosis of 4R-tauopathies was made according to the revised Armstrong Criteria of probable CBS^37^ or the Movement Disorders Society criteria of possible/probable PSP or possible PSP with predominant CBS.^35^ Inclusion criteria were age > 45 years at the time of inclusion, and stable pharmacotherapy for at least one week before the PET examination. Exclusion criteria were severe neurological or psychiatric disorders other than AD-continuum or 4R-tauopathies. Regional quantification in predefined regions of interest in AD continuum and PSP patients as well as in healthy controls (comprising 59% of the current sample) was previously published.^32^ Healthy controls had no evidence of cognitive impairment following a neuropsychological battery which included the ADAS-Cog, a CDR score of 0, no family history of AD or neurological disease associated with dementia and no objective motor symptoms. PET data analyses were approved by the local institutional review board of the LMU Munich (application numbers 17-569 & 19-022) under consideration of the Helsinki Declaration of 1975 (and as revised in 1983). All participants provided written informed consent prior to the PET scan. The β-amyloid status was obtained by PET ([^18^F]florbetaben or [^18^F]flutemetamol) or cerebrospinal fluid assessment. β-amyloid-positive clinical PSP/CBS as well as β-amyloid-negative clinical AD cases were excluded from the analysis since the clinically predicted tau isoform would have been uncertain in these cases. To allow comparison of 3/4R and 4R tauopathies in matching brain regions, only visually rated [^18^F]PI-2620-positive cases in cortical brain areas were included into the analysis of patients. One expert reader assessed the [^18^F]PI-2620 distribution volume ratio (DVR) maps in 3 D mode using standardized settings (lower/upper DVR threshold 1.0/1.5; cold color scale, overlay on an MRI standard template in the Montreal Neurology Institute (MNI) space. The reader evaluated binding in the frontal, parietal, temporal and occipital cortex with knowledge of the pattern in healthy controls. The intention of conservative judgement of cortical binding in DVR maps was to avoid inclusion of any artificial clusters. The reader was aware of the clinical diagnosis. Subcortical [^18^F]PI-2620-positivity was no prerequisite for inclusion. Figure 1(a) illustrates the selection process.

### Acquisition, reconstruction and image harmonization

[^18^F]PI-2620 PET imaging was performed in a full dynamic setting (0–60 min p.i.) on different scanners (Munich: Siemens Biograph True point 64 PET/CT & Siemens mCT, Siemens, Erlangen, Germany; New Haven: Siemens ECAT EXACT HR+, Siemens, Erlangen, Germany; Melbourne: Philips Gemini TF 64 PET/CT, Eindhoven, The Netherlands) at three specialized neuroimaging sites using the established standard scan protocol of each center for brain PET imaging. The injected bolus dose was 217 ± 53 MBq (range: 178–334 MBq). Details on all scanners, as well as acquisition and reconstruction parameter are provided in the Supplement of our previous study.^32^ Dynamic emission recordings were framed into 6x30s, 4x60s, 4x120s and 9x300s. Data from Hofmann phantoms were used to obtain scanner specific filter functions which were then consequently used to generate images with a similar resolution (FWHM: 9 × 9 × 10 mm), following the ADNI image harmonization procedure.^40^ All dynamic datasets were visually inspected for motion (∼5 mm threshold) and automatically corrected for motion of more than 5 mm using the motion correction tool in PMOD (V3.9 PMOD technologies Basel, Switzerland). In brief, this tool performs coregistration of individual PET frames to each other to guarantee a precise regional overlap of all frames in a multi-frame dataset.

### Image post-processing

#### [^18^F]PI-2620 template generation

An [^18^F]PI-2620 PET template was generated independently of this investigation^32^ to allow inclusion of patients not eligible to an MRI (i.e. pacemaker, metal implants). In brief, 20 randomly chosen datasets of PSP patients, disease controls and healthy controls, which all had a T1w 3 D MRI sequence were automatically processed by the PNEURO pipeline^20^ to obtain [^18^F]PI-2620 images in the MNI space. Frames between 30 and 60 minutes p.i. were summed and scaled by the global mean, followed by averaging all 20 cases to an [^18^F]PI-2620 template in the MNI space. The template already showed robust performance in cases with high and low [^18^F]PI-2620 positivity due to the well-defined landmarks of the tracer in the 30-60 minutes p.i. window.^32^

#### Spatial *normalization*

All 50 dynamic [^18^F]PI-2620 PET datasets (39 patients and 11 healthy controls) were transformed to the MNI space using the non-linear brain normalization of the summed 30–60 min frames to the established [^18^F]PI-2620 PET template as described previously.^32^ In brief, automatized brain normalization settings in PMOD included nonlinear warping, 8 mm input smoothing, equal modality, 16 iterations, frequency cutoff 3, regularization 1.0, and no thresholding. The transformation was saved and applied to the full dynamic [^18^F]PI-2620 PET datasets to assure a minimum of interpolation.

#### Quantification

In preparation of the cluster-based analysis by various kinetic models, [^18^F]PI-2620-positive clusters had to be defined by a standardized approach. Thus, we applied the previously established quantification approach for generation of parametric images.^32^ The multilinear reference tissue model 2 (MRTM2^41^) was used to calculate distribution volume ratios (DVR; DVR = BPND + 1) images of each full dynamic dataset. The previously evaluated cerebellar reference tissue^32^ (detailed illustration in Supplemental Figure 1), excluding the dentate nucleus and the central cerebellar white matter as well as the superior and the posterior cerebellar layers (d = 1.5 cm each), served as a reference region.

#### Generation of Z-score maps

MRTM2 images of eleven healthy controls were used to calculate average and standard deviation maps of controls. All MRTM2 maps of the 39 patients were processed by the following formula using the PMOD image algebra tool to generate Z-score maps of individual patients:

$$\begin{array}{l} Patient\;Z\;score\;map\\ \;=\frac{\left\{\begin{array}{l} Patient\;MRTM2\;DVR\;map\\ \;-Average\;HC\;MRTM2\;DVR\;map \end{array}\right\}}{\left\{\begin{array}{l} S\tan dard\;deviation\;HC\;\\ \;MRTM2\;DVR\;map \end{array}\right\}} \end{array}$$

### Data analysis

#### Definition of cortical and subcortical [^18^F]PI-2620-positive clusters

The rationales of a cluster-based analysis were robust kinetic modeling in volumes large enough in relation to the resolution of the PET system and evaluation in regions that likely comprise tau in the patient. Thus, we set conservative thresholds for the cluster size and [^18^F]PI-2620-positivity. Furthermore, we used predefined brain volumes (defined by lobes and basal ganglia compartments) for anatomical cluster definition. Predefined regions of the Hammers atlas and the atlas of the basal ganglia^42,43^ were used for tissue classification in Z-score maps. We defined frontal, parietal, temporal, and occipital lobes as cortical target regions as well as the putamen and the globus pallidus as subcortical target regions. Within each target region, clusters exceeding 20 voxels (volume: 160 mm³, corresponding to a 5.0 × 5.0 × 6.4 mm sphere) of elevated DVR (≥2.5 SD vs. eleven healthy controls) were classified as [^18^F]PI-2620-positive. The strict threshold was used to avoid inclusion of voxels with artificially high values, caused by spillover from compartments adjacent to the brain (i.e. venous sinuses). The cluster volume was recorded. Figure 1(b) illustrates the definition of these assumed tau-positive clusters. All further analyses were based on [^18^F]PI-2620 binding in these clusters.

#### Kinetic modeling and extraction of [^18^F]PI-2620 binding parameters

We evaluated different kinetic modeling approaches to test for robustness of similar parameters among these models. Non-invasive kinetic modelling approaches (simplified reference tissue modeling approaches 1 & 2 (SRTM & SRTM2) and MRTM2) were separately ran for all [^18^F]PI-2620-positive clusters with the cerebellar reference tissue by PMOD. R1 values as a surrogate of delivery as well as k2 & k2a efflux rate parameters as surrogate of dissociation from the target were extracted from SRTM and SRTM2. A lower limit of 0.006/min and an upper limit of 0.6/min were set for k2a and clusters reaching these limits were excluded. DVR values as a parameter for overall binding magnitude were extracted from all models and reported for MRTM2. A lower limit of 0 and an upper limit of 5 were set for DVR and clusters reaching these limits were excluded. Delivery, efflux rate and DVR deriving from different models were correlated with each other to test for their robustness. k2’ and t* (equilibration time) were fixed across models. In addition, late binding phase uptake as a surrogate for the binding magnitude was obtained from static images between 30 and 60 minutes p.i. (SUVR_30-60_). The cluster-to-cerebellum SUVRs were computed in each frame for a model-independent evaluation of the signal kinetic. Linear functions were calculated for all intervals i-60 min p.i. starting at 9 min p.i. (i.e. 9–60 min p.i., 11–60 min p.i., etc.) and the slopes were used for characterizing the post-perfusion binding phase.

### Statistics

A sample size of 6 patients per group (3/4R and 4R) gave a power of >0.8 when using previous DVR data ^32^ for sample size estimation (α = 0.05; cortex 3/4R: mean 1.17 SD 0.20; cortex 4R: mean 0.89 SD 0.07) by G*Power (V3.1.9.2, Kiel, Germany). Regional cluster volumes, R1-values, k2 & k2a values, DVR, SUVR_30-60_, and the slope were compared between 3/4R and 4R tauopathies by a two-sample t-test. For comparisons between different tau-positive regions, a one-way analysis of variance was applied (>2 comparison groups). All analyses were conducted using SPSS 25 (V25, IBM, Ehningen, Germany) and GraphPad Prism 8 (GraphPad Software, San Diego, USA). Receiver operating characteristics (ROC) were used to calculate the area under the curve (AUC) for discriminating 3/4R vs. 4R tauopathy by the above read-outs. Furthermore, a principal component analysis (PCA) of five parameters (R1 with highest AUC, k2 & k2a with highest AUC, DVR, SUVR_30-60_, slope with highest AUC) was performed in SPSS and also subject to ROC evaluation. Linear relationship of the data was tested by a correlation matrix and items with a correlation coefficient <0.3 were discarded. The Kaiser-Meyer-Olkin (KMO) measure and Bartlett’s test of sphericity were used to test for sampling adequacy and suitability for data reduction. Components with an Eigenvalue > 1.0 were extracted and a varimax rotation was included.

### Data availability

Data of this manuscript are available through contacting the corresponding author upon reasonable request.

## Results

### Demographics and [^18^F]PI-2620-positive clusters in clinically diagnosed 3/4R and 4R tauopathies

10 patients with a clinically diagnosed 3/4R tauopathy (10 AD continuum) and 29 patients with a clinically diagnosed 4R tauopathy (14 CBS, 15 PSP) according to current diagnostic criteria, all with a discernible [^18^F]PI-2620 cortical signal in visual evaluation, were enrolled for this evaluation. 3/4R tauopathy patients (65.7 ± 10.1 y; 70% female), 4R tauopathy patients (71.0 ± 7.2 y; 38% female) and healthy controls (67.5 ± 6.7 y; 73% female) did not differ significantly in age.

Suitability of the reference tissue was validated by similarity of time-activity-curves, SUV (3/4R: 0.36 ± 0.07 g/ml; 4R: 0.37 ± 0.09 g/ml; healthy controls: 0.38 ± 0.08 g/ml), and their coefficients of variation (3/4R: 18%; 4R: 25%; healthy controls: 21%) in the study groups (Supplemental Figure 2). The frequency and regional volumes of cortical and subcortical [^18^F]PI-2620-positive clusters in 3/4R and 4R tauopathies are shown in Table 1. The 3/4R tauopathy group showed [^18^F]PI-2620-positive clusters in nearly all cortical regions, whereas the frequency of [^18^F]PI-2620-positive clusters in cortical regions was lower in 4R cases. The 4R tauopathy group showed a higher percentage of [^18^F]PI-2620-positive subcortical clusters when compared to 3/4R cases (77% vs. 35%, p < 0.0001).

**Table 1.** Frequency and regional volume of target clusters in 3/4R (10 patients) and 4R tauopathies (29 patients).

	3/4R tauopathy		4R tauopathy		
	Volume (mean ± SD (ccm))	n	Volume (mean ± SD (ccm))	n	P-value (volume)
Cortical	54.4 ± 51.6	79	4.8 ± 11.1	167	<0.0001
Frontal L	76.2 ± 85.4	10	6.4 ± 14.5	26	0.0002
Frontal R	79.2 ± 92.0	10	8.7 ± 19.3	25	0.0007
Occipital L	41.8 ± 27.1	10	6.4 ± 10.2	15	0.0001
Occipital R	38.1 ± 30.7	10	4.7 ± 8.9	18	0.0002
Parietal L	45.2 ± 34.7	10	3.4 ± 7.0	20	<0.0001
Parietal R	50.0 ± 35.0	9	3.0 ± 6.6	20	<0.0001
Temporal L	55.2 ± 18.1	10	3.0 ± 5.7	21	<0.0001
Temporal R	48.8 ± 34.4	10	2.2 ± 4.2	22	<0.0001
Subcortical	1.8 ± 1.9	14	2.0 ± 1.7	89	0.6871
Putamen L	1.3 ± 1.3	4	2.8 ± 1.8	24	0.1245
Putamen R	2.5 ± 2.4	7	2.8 ± 1.8	27	0.6802
G. Pallidus L	–	0	0.8 ± 0.3	17	–
G. Pallidus R	0.9 ± 0.8	3	1.0 ± 0.6	21	0.8076

Note: P values derive from an unpaired t-test for the comparison of regional cluster volumes between 3/4R and 4R tauopathies. L: left; R: right; SD: standard deviation.

The volume of combined cortical [^18^F]PI-2620-positive clusters was larger for the 3/4R tauopathy group when compared to the 4R tauopathy group (54.4 ± 51.6 mm³ vs. 4.8 ± 11.1 mm³, p < 0.0001), whereas there was no volume difference between both groups for combined subcortical clusters (1.8 ± 1.9 mm³ vs. 2.0 ± 1.7 mm³, p = 0.687).

### Kinetic modeling parameters in comparison between 3/4R and 4R tauopathies

To estimate kinetic properties of [^18^F]PI-2620, R1 and k2 & k2a-values were compared between groups of 3/4R and 4R tauopathies and between cortical and subcortical brain regions (Table 2). 88% of the MRTM2 defined [^18^F]PI-2620-positive clusters resulted in modeling parameters within the defined threshold ranges for the applied kinetic models. Delivery, efflux and binding parameters of different models correlated highly with each other (delivery: all R > 0.9; k2 & k2a: all R > 0.99).

**Table 2.** Delivery (R1) and efflux (k2 & k2a) parameters of [^18^F]PI-2620-positive clusters in cortical and subcortical brain areas.

	SRTM R1 (unitless ± SD)	SRTM2 R1 (unitless ± SD)	SRTM k2 (1/min ± SD)	SRTM k2a (1/min ± SD)	SRTM2 k2a (1/min ± SD)
Cortical PI-2620-positive clusters					
3/4R	0.83 ± 0.10	0.83 ± 0.10	0.06 ± 0.07	0.05 ± 0.07	0.05 ± 0.07
4 R	0.92 ± 0.21	0.88 ± 0.20	0.17 ± 0.21	0.15 ± 0.19	0.15 ± 0.19
p value (3/4R vs. 4R)	0.0007	0.0380	<0.0001	<0.0001	<0.0001
HC	0.87 ± 0.11	0.83 ± 0.08	0.17 ± 0.23	0.19 ± 0.26	0.07 ± 0.17
Subcortical PI-2620-positive clusters					
3/4R	1.02 ± 0.15	1.02 ± 0.15	0.25 ± 0.20	0.19 ± 0.16	0.19 ± 0.16
4 R	0.96 ± 0.14	0.96 ± 0.14	0.21 ± 0.14	0.16 ± 0.11	0.16 ± 0.11
p value (3/4R vs. 4R)	0.1515	0.1431	0.3657	0.2924	0.2698
HC	0.77 ± 0.10	0.77 ± 0.09	0.14 ± 0.14	0.16 ± 0.17	0.07 ± 0.10
3/4 R tauopathies					
Cortical	0.83 ± 0.10	0.83 ± 0.10	0.06 ± 0.07	0.05 ± 0.07	0.05 ± 0.07
Subcortical	1.02 ± 0.15	1.02 ± 0.15	0.25 ± 0.20	0.19 ± 0.16	0.19 ± 0.16
p value (cortical vs. subcortical)	<0.0001	<0.0001	<0.0001	<0.0001	<0.0001
4R tauopathies					
Cortical	0.92 ± 0.21	0.88 ± 0.20	0.17 ± 0.21	0.15 ± 0.19	0.15 ± 0.19
Subcortical	0.96 ± 0.14	0.96 ± 0.14	0.21 ± 0.14	0.16 ± 0.11	0.16 ± 0.11
p value (cortical vs. subcortical)	0.1019	0.0018	0.1545	0.8293	0.8293

Note: Values of healthy controls (HC) were extracted from atlas regions and serve for an orienting comparison, whereas values of 3/4R and 4R tauopathies were derived from [^18^F]PI-2620 positive clusters.

**Figure 2.**
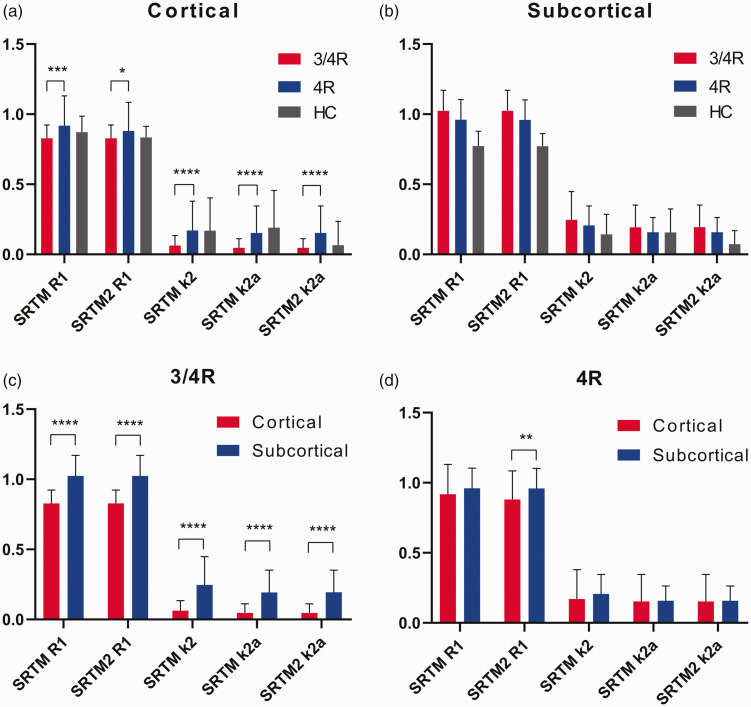
Kinetic modelling parameter of delivery and efflux of [^18^F]PI-2620 in comparison of clinically diagnosed 3/4 R (n = 10) and 4 R (n = 29) tauopathies. Delivery and efflux of 4 R tauopathy patients were higher in [^18^F]PI-2620-positive cortical clusters (a) but not different in subcortical clusters (b). Subcortical clusters showed higher delivery and/or efflux parameters when compared to cortical clusters regardless of the patient group (c, d). Data of healthy controls (HC) were extracted from atlas regions and serve for an orienting comparison. Error bars represent standard deviation of study groups. *p < 0.05, **p < 0.01, ***p < 0.001, and ****p < 0.0001 indicate significant differences between 3/4 R and 4 R tauopathies as assessed by an unpaired Student’s t-test.

In cortical regions, [^18^F]PI-2620-positive clusters of 4R tauopathy cases had higher R1 (all p < 0.05) and k2 & k2a (all p < 0.0001) values when compared to 3/4R tauopathy cases, suggesting a faster tracer delivery and efflux from the target in the presence of 4R tau (Figure 2(a)). Subcortical clusters did not show differences of R1 and k2 & k2a values between 4R and 3/4R tauopathies, indicating similar delivery and efflux of [^18^F]PI-2620 to subcortical regions for both types of tau (Figure 2(b)).

Next, we asked whether [^18^F]PI-2620 binding characteristics are different between cortical and subcortical brain regions. 3/4R tauopathy subjects had elevated R1 and elevated k2 & k2a values in subcortical areas, when compared to [^18^F]PI-2620-positive cortical clusters (Figure 2(c)). Within 4R-tauopathies, R1 of SRTM2 was lower in cortical clusters when compared to subcortical clusters (0.88 ± 0.20 vs. 0.96 ± 0.14, p = 0.0018; Figure 2(d)). We observed the lowest k2 & k2a values in cortical clusters of 3/4R tauopathy cases.

### Binding magnitude in comparison between 3/4R and 4R tauopathies

[^18^F]PI-2620 binding was assessed for the full 60-minutes scan (DVR) and a binding surrogate was obtained for the late uptake phase (SUVR_30-60_). 3/4R tauopathy cases showed significantly higher DVR values in all cortical [^18^F]PI-2620-positive clusters when compared to 4R tauopathies (Table 3).

**Table 3.** Distribution volume ratios (DVR) and standardized uptake value ratios between 30 and 60 minutes post injection (SUVR_30-60_) of [^18^F]PI-2620-positive clusters in cortical and subcortical brain areas.

	DVR						SUVR_30–60_							
	3/4R tauopathy		4R tauopathy		P	Healthy controls		3/4R tauopathy		4R tauopathy		p	Healthy controls	
	Mean ± SD	n	Mean ± SD	n	Mean ± SD	n		Mean ± SD	n	Mean ± SD	n		Mean ± SD	n
Cortical	1.38 ± 0.18	79	1.13 ± 0.10	167	<0.0001	0.94 ± 0.05	88	1.76 ± 0.34	79	1.31 ± 0.20	167	<0.0001	1.05 ± 0.06	88
Frontal L	1.27 ± 0.12	10	1.11 ± 0.06	26	<0.0001	0.90 ± 0.05	11	1.58 ± 0.26	10	1.26 ± 0.11	26	<0.0001	1.01 ± 0.06	11
Frontal R	1.24 ± 0.17	10	1.10 ± 0.07	25	0.0012	0.90 ± 0.04	11	1.54 ± 0.32	10	1.24 ± 0.14	25	0.0005	1.00 ± 0.05	11
Occipital L	1.45 ± 0.19	10	1.20 ± 0.09	15	0.0002	0.98 ± 0.03	11	1.84 ± 0.41	10	1.38 ± 0.27	15	0.0023	1.10 ± 0.05	11
Occipital R	1.43 ± 0.19	10	1.14 ± 0.05	18	<0.0001	0.99 ± 0.03	11	1.83 ± 0.37	10	1.30 ± 0.14	18	<0.0001	1.09 ± 0.04	11
Parietal L	1.44 ± 0.23	10	1.09 ± 0.06	20	<0.0001	0.93 ± 0.05	11	1.84 ± 0.39	10	1.28 ± 0.11	20	<0.0001	1.04 ± 0.05	11
Parietal R	1.43 ± 0.21	9	1.07 ± 0.08	20	<0.0001	0.94 ± 0.05	11	1.86 ± 0.39	9	1.25 ± 0.14	20	<0.0001	1.04 ± 0.04	11
Temporal L	1.42 ± 0.09	10	1.20 ± 0.13	21	<0.0001	0.95 ± 0.05	11	1.83 ± 0.17	10	1.38 ± 0.29	21	<0.0001	1.06 ± 0.06	11
Temporal R	1.40 ± 0.12	10	1.18 ± 0.15	22	0.0003	0.96 ± 0.05	11	1.79 ± 0.27	10	1.40 ± 0.29	22	0.0011	1.07 ± 0.05	11
Subcortical	1.22 ± 0.17	14	1.32 ± 0.09	89	0.0012	0.94 ± 0.09	44	1.16 ± 0.25	14	1.38 ± 0.17	89	<0.0001	0.98 ± 0.14	44
Putamen L	1.24 ± 0.20	4	1.35 ± 0.08	24	0.0569	0.89 ± 0.06	11	1.15 ± 0.26	4	1.37 ± 0.15	24	0.0200	0.89 ± 0.09	11
Putamen R	1.18 ± 0.20	7	1.30 ± 0.12	27	0.0539	0.87 ± 0.06	11	1.14 ± 0.30	7	1.29 ± 0.18	27	0.0978	0.88 ± 0.09	11
G. Pallidus L	–	0	1.32 ± 0.06	17	–	0.97 ± 0.07	11		0	1.46 ± 0.17	17	–	1.05 ± 0.08	11	
G. Pallidus R	1.28 ± 0.07	3	1.31 ± 0.08	21	0.5417	1.04 ± 0.07	11	1.24 ± 0.17	3	1.44 ± 0.14	21	0.0337	1.12 ± 0.11	11

Note: Values of healthy controls (HC) were extracted from atlas regions and serve for an orienting comparison, whereas values of 3/4 R and 4 R tauopathies were derived from [^18^F]PI-2620 positive clusters. L: left; R: right.

SUVR_30-60_ were consistently higher in [^18^F]PI-2620-positive cortical clusters of 3/4R tauopathy patients when compared to 4R tauopathy patients. Subcortical areas indicated a higher binding magnitude for [^18^F]PI-2620-positive clusters in patients with 4R tauopathies when compared to 3/4R tauopathies.

### Slope of binding during the post-perfusion phase

**Figure 3.**
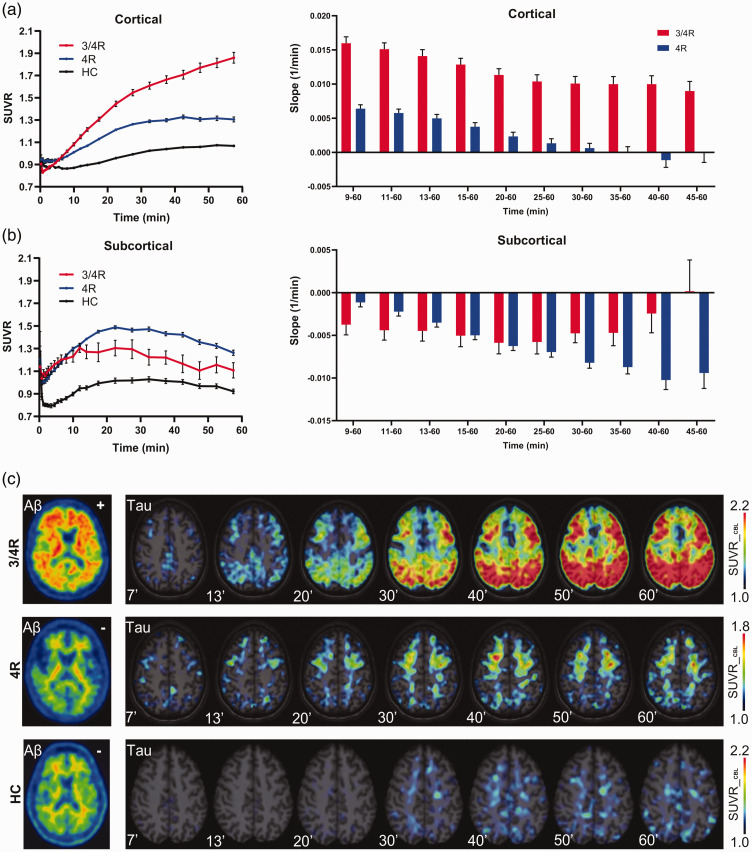
(a,b) Time–SUVR curves in the comparison of clinically diagnosed 3/4R (n = 10), 4R (n = 29) tauopathies, and healthy controls (HC) together with the slope of different time intervals during the post-perfusion phase. Time–SUVR values of HC were extracted from atlas regions, whereas time–SUVR values of 3/4R and 4R tauopathies were derived from [^18^F]PI-2620 positive clusters. (c) Native β-amyloid-PET images together with tau-PET SUVR images of cortical areas for exemplary cases of patients with 3/4R and 4R tauopathies and a healthy control in different time frames of the post-perfusion phase. Axial slices show [^18^F]PI-2620 SUVR upon an MRI atlas. Error bars represent standard error (SEM) of single frame values and intervals in study groups.

[^18^F]PI-2620 time–SUVR curves of both diagnostic groups clearly separated over time for all cortical brain areas but not for subcortical regions (Figure 3(a) and (b)). Time-SUVR curves of 3/4R subjects showed a continuous increase in all cortical areas over nearly all frames, whereas cortical time-SUVR curves of 4R subjects reached a plateau at 30 min after injection for parietal cortex or even decreased from 30 min after injection for frontal cortex (Supplemental Figure 3). In subcortical regions, time–SUVR curves of 3/4R subjects did not separate from those of 4R subjects in both putamen and globus pallidus and showed a similar decrease from 30 to 60 min p.i. (Supplemental Figure 3).

**Figure 4.**
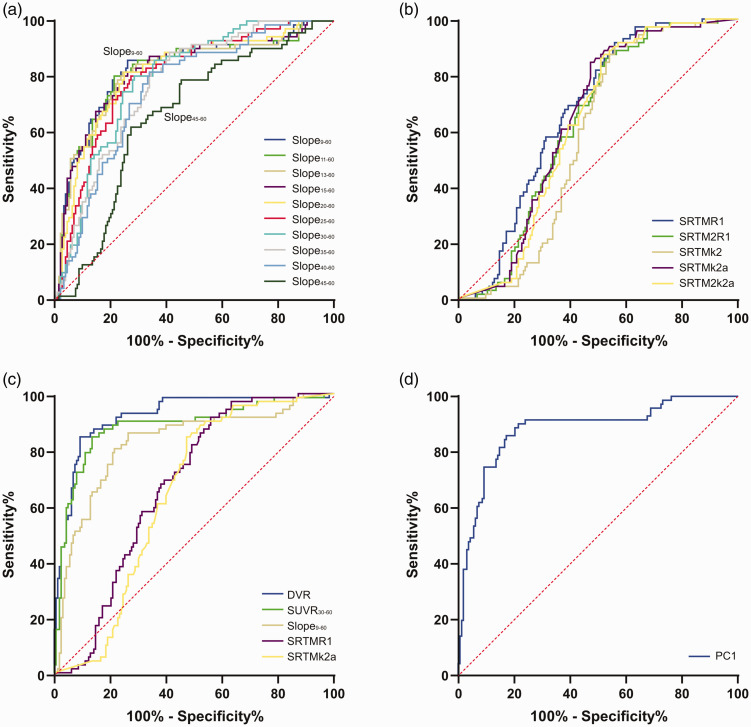
ROC comparison for differentiation of 3/4 R (n = 10) tauopathies from 4 R (n = 29) tauopathies. ROC curves are shown for (a) slopes in different intervals of the post-perfusion phase, (b) delivery and efflux parameters deriving from different modelling approaches, (c) in contrast of all single study read outs, and (d) for the principal component 1 of all read outs. Only read outs of cortical clusters were used for the ROC analysis.

Based on these findings we plotted single frame [^18^F]PI-2620 SUVR as a linear function of time between 9 to 60 min and extracted the slope of the equation for each single cluster to enable a quantitative comparison between 3/4R and 4R tauopathies. The slope was nearly exclusively positive in cortical regions and nearly exclusively negative in subcortical regions for all evaluated time intervals, regardless of the assumed tau isoform (Figure 3(a) and (b)). Strikingly, in cortical regions the slope of 3/4R tauopathy cases was much steeper when compared to 4R tauopathy cases for all time intervals, also discernible by visual interpretation of single cases (Figure 3(c)). The strongest discrimination of the slope between 3/4R and 4R tauopathies was observed for the time interval between 9 and 60 min (slope_9-60_: 0.016/min ± 0.008/min vs. 0.006/min ± 0.007/min, p < 0.0001, AUC: 0.824; Figure 4(a)). In the direct contrast to cortical areas, subcortical regions only indicated minor differences of the slope between 3/4R and 4R tauopathies, comprising stronger negative values in 4R tauopathies for late time intervals (i.e. slope_40-60_: −0.002/min ± 0.008/min vs. −0.010/min ± 0.011/min; p = 0.010).

### Merged value of all kinetic modeling parameters for the discrimination of 3/4R and 4R tauopathies

A comparison of kinetic modeling parameters, DVR, SUVR, and slopes of 3/4R and 4R tauopathies were conducted to estimate the feasibility of predicting a 3/4R tauopathy by [^18^F]PI-2620 PET. To this end we performed a ROC analysis and computed AUC of all evaluated parameters.

AUCs of the different slope intervals were highest for the longest interval (slope_9-60,_ AUC: 0.824) and decreased with elapsed scan duration (slope_45-60,_ AUC: 0.714; Figure 4(a)). AUCs for delivery and efflux parameters from different kinetic models were similar (Figure 4(b)). ROC of DVR (AUC: 0.922) and SUVR_30-60_ (AUC: 0.906) resulted in a stronger prediction of a 3/4R tauopathy in cortical [^18^F]PI-2620-positive clusters when compared to the best slope_9-60_ (AUC: 0.824), the best delivery (AUC: 0.654) and the best efflux discriminations (AUC: 0.716; Figure 4(c)).

The principal component analysis resulted in a KMO sampling adequacy of 0.539 and Bartlett’s test of sphericity was significant (p < 0.001). We found two principal components with an Eigenvalue > 1.0 (principal component 1: 2.729, principal component 2: 1.491) which explained 84.4% of the variance. The component matrix for the principal component 1 was 0.980 for SUVR_30-60_, 0.908 for DVR and 0.901 for slope_9-60_. The component matrix for the principal component 2 was 0.879 for R1_SRTM_ and 0.877 for k2a_SRTM_. AUC for the discrimination of 3/4R and 4R tauopathies by principal component 1 was 0.899 (Figure 4(d)) and 0.669 for principal component 2.

## Discussion

This is the first study investigating the kinetic and binding characteristics of [^18^F]PI-2620 in different brain regions of clinically diagnosed 3/4R and 4R tauopathies by non-invasive reference tissue modelling. We demonstrate that [^18^F]PI-2620-positive clusters of clinically diagnosed 4R tauopathy cases have higher delivery and efflux parameters when compared to similar clusters in clinically diagnosed 3/4R tauopathies. This finding was also reflected by lower slopes of time-activity-ratio curves between 9 and 60 minutes after tracer injection for cortical clusters in 4R tauopathies when compared to 3/4R tauopathies. We consider this effect as the *in vivo* correlate of the reported lower [^18^F]PI-2620 affinity to 4R tau *in vitro*. Binding characteristics in subcortical brain regions are dominated by higher delivery and efflux when compared to cortical regions. Ultimately, the assumed presence of 3/4R and 4R tau in the individual brain can be differentiated by the kinetic and binding characteristics of [^18^F]PI-2620.

In this study, we report kinetic modeling results for dynamic 0- to 60-min [^18^F]PI-2620 PET scans in patients with 3/4R and 4R tauopathies. We observed enhanced R1 and k2 & k2a values in cortical [^18^F]PI-2620-positive clusters of the 4R tauopathy group compared to the 3/4R tauopathy group (Table 2). The stronger efflux of the tracer from the target in 4R tauopathies was visually confirmed by clearly separated cortical time-SUVR curves of 3/4R- and 4R-tau cases with increasing scan duration and the visual inspection of single frame images. In particularly, SUVR of all cortical regions of 3/4R-tau cases kept increasing over time, while time-SUVR curves of parietal and frontal cortices of 4R tau-cases reached a plateau or had a slight decrease in the late phase (Supplemental Figure 3). This indicates that 4R-tau cases have a slightly higher delivery rate from blood to assumed tau-positive cortical target regions and a significantly higher clearance rate to blood. The higher efflux parameters suggest a less stable binding of [^18^F]PI-2620 to 4R tau in comparison to 3/4R tau which is in line with the lower affinity of this tracer to synthetic 4R tau fibrils when compared to 3/4R fibrils.^25^ The radioligand [*^18^* F]flortaucipir, aka [^18^F]AV-1451, is the by far most widely studied tau-PET tracer to date. Similar to our study, one previous study clearly demonstrated that AV-1451 has unique *in vivo* binding properties for assumed AD-like tau pathology among MAPT mutation carriers.^44^ Besides the consistent *in vivo* retention of [^18^F]AV-1451, in vitro autoradiography suggest that [^18^F]AV-1451 displays strong binding affinity to paired helical filaments tau in Alzheimer’s disease, but exhibits low affinity to tau aggregates primarily made of straight tau filaments in these non-Alzheimer tauopathies.^5,45,46^ Similarly, another study has shown poor binding of [^18^F]AV-1451 to non-AD tauopathies that have an accumulation of either 3R or 4R tau.^47^ The reasons for the binding differences of [^18^F]PI-2620 and [^18^F]AV-1451 for 3/4R and 4R tau isoforms are not yet understood. A potential explanation could be based on a different configuration of tracer binding pockets in the different forms of tau aggregates. Cryo-electron microscopy (cryoEM) studies recently showed that paired helical filaments and straight filaments of 3/4R tau from AD contained the same C-shape protofibril structure whereas the 4R tau-structure obtained from CBD was distinctly different.^48^ Detailed analysis of the tracer binding sites at the ultrastructure level using cryoEM could potentially elucidate the mechanisms behind the different specificity of [^18^F]PI-2620 and other tau tracers to different isoforms and filaments of tau. Kroth and coworkers recently described the preclinical comparison of [^18^F]AV1451 and [^18^F]RO948 with other fluoropyridine regioisomers.^25^ They found that different regioisomers gave rise to a remarkably diverse set of characteristics with regard to their binding affinity for AD and PSP tau, off-target binding to beta-amyloid and monoamine oxidases A or B as well as global brain uptake and washout in mice. Based on this preclinical comparison, [^18^F]PI-2620 was selected as clinical candidate with optimized binding to AD but also to PSP.

Tracer kinetics and binding characteristics of [^18^F]PI-2620 in clusters of subcortical areas were not different between clinically diagnosed 3/4R and 4R tauopathies, Since inverted U-shape time-SUVR curves of [^18^F]PI-2620 were also observed in healthy controls,^32^ we speculate that the effect of binding affinity to tau deposition is dominated by the general tracer kinetics in subcortical regions. Our findings of higher initial uptake and much faster clearance in the putamen and the globus pallidus in comparison to cortical regions are similar to reports on kinetic characteristics of [^18^F]AV-1451.^49–51^ Keeping in mind that our [^18^F]PI-2620 study analyzed assumed tau-positive clusters and earlier [^18^F]AV-1451 studies used predefined brain regions, the increasing time-SUVR curves for AD subjects and the inverted U-shape of time-SUVR curves for 4R tauopathy subjects of our study correspond well with the aforementioned studies. Similar to our obtained delivery parameter R1, k1 of [^18^F]AV-1451 was highest in the putamen and lower for cortical regions.^50^ Another study on [^18^F]AV-1451 showed that the putamen of AD subjects has a higher SUVR at the initial wash-in stage, reaching a steady-state condition at 40–50 min post-injection.^52^ Generally, lower subcortical SUVR of [^18^F]PI-2620 in comparison to [^18^F]AV-1451 might suggest lower off-target binding in the basal ganglia, which would be an improvement of next-generation tau-PET imaging.^14,32^ These findings indicate that some subcortical binding of both tracers is probably caused by yet unknown mechanisms potentially involving a secondary binding site with different kinetics or extra off-target binding in addition to the specific binding to tau aggregates. Another potential mechanism could rely on different capillary permeability between brain regions, supposing a higher capillary permeability in subcortical areas, which implies an increased extraction through the blood-brain barrier, in the putamen and the globus pallidus but not in the cerebral cortex.^53^ Although we cannot rule out non-specific binding or off-target binding, the clear basal ganglia [^18^F]PI-2620 binding pattern of some AD cases (∼30%) could be explained by a recent Japanese autopsy study, which reported on the presence of tau in the basal ganglia of AD cases.^54^ Our current data support the hypothesis that even low deposition of 3/4R tau in the basal ganglia of AD could cause a higher PET signal when compared to strong 4R tau deposition in the basal ganglia of PSP cases.

The main focus of our study was to translate non-invasive kinetic modelling of [^18^F]PI-2620 PET data into an application for differential diagnosis. The differentiation of tauopathies is important since there are overlapping clinical syndromes in presence of different tau isoforms. for example, there is an underestimated overlap of the phenotype (amnestic mild cognitive impairment) between AD and argyrophilic grain disease at early stages^34^ and AD-CBS and 4R CBS both manifest in motor areas of the frontal lobe.^55^ As an internal validation of our data, we applied different non-invasive approaches of simplified and multilinear kinetic modelling and observed highly similar parameters of binding characteristics between them. We suggested earlier that the development of selective 4R tracers could facilitate the differentiation of 4R-tauopathies not only from healthy controls but also from AD cases with predominant presence of 3/4R tau.^31^ Despite the different magnitude and spatial extent of brain tau deposition, [^18^F]PI-2620 binding properties were already able to differentiate 3/4R from 4R tauopathies. Importantly, this was not only true for regions exclusively assumed tau-positive in either of 3/4R or 4R tauopathies but also in overlapping regions, i.e. the frontal cortex. Thus, although [^18^F]PI-2620 is likely not specific to 3/4R- or 4R tau, the AUC of kinetic modeling parameter and binding characteristics suggest feasibility of differential diagnosis between 3/4R and 4R tauopathies. We implemented a principal component analysis which did not indicate higher AUC values for discrimination when compared to single parameters of binding magnitude. This is probably related to the composition of our patient cohort, which includes a high proportion of severe cases in the 3/4R group. Thus, a potential additive value of assessing the principal component of all parameters or the slope of the late binding phase needs to be explored by future cases with similar binding magnitude. The rationale for the assessment of the slope in the late binding phase was based on the possibility to assess this parameter also in a late imaging window between 30 and 60 minutes p.i. While the slope between 9 and 60 minutes p.i. separated better between 3/4R and 4R tauopathy patients, the discrimination was still present for the slope between 30 and 60 minutes p.i., providing an alternative when economic reasons or patient compliance do not allow dynamic scanning. The methodology could be of interest for target engagement when future anti-tau treatments are specific for 3/4R tau or 4R isoforms. The assessment of the early phase as a surrogate biomarker of neuronal injury ^56^ adds another part to the toolbox of this tracer when dynamic imaging is performed. The current dataset was too small to evaluate multi-parameter classification approaches or artificial intelligence for prediction of 3/4R and 4R tauopathy cases but such methods are likely interesting when larger [^18^F]PI-2620 cohorts will be available.

Among the limitations of the study we focused on non-invasive kinetic modeling and our data are lacking full quantification by arterial blood sampling. Thus, we are not able to test for effects of different tracer plasma levels and tracer metabolism of [^18^F]PI-2620 in the investigated subjects. Furthermore, our assumptions of modeling parameters rely on simplified reference region methodology which can be subject to bias and variability when compared to arterial input measures. However, due to the rare incidence and severe disability of 4R tauopathy patients our current dataset likely provides a good compromise between clinical feasibility and accuracy. To account for the prerequisites of reference tissue modeling we chose the previously established cerebellar reference tissue excluding the dentate nucleus to generate a compartment with assumed negligible specific binding in 3/4R and 4R tauopathies.^32^ We used a simple linear fit for the slope of the late binding phase and we note that this fit is imperfect for cases with an inverted U-shape of their time-activity-curve. While more flexible fits could further improve the methodology, the proposed approach will more likely be transferable to clinical scenarios. Clinical applicability was also the reason to not apply partial volume effect correction in the current study design. This is a limitation, as atrophy is related to lower fit values of R1 and lower [^18^F]PI-2620 binding. We used the slope of the time-activity-ratio-curve to characterize the late binding phase and also found a strong discrimination between 3/4R and 4R tauopathies. This index should be less affected by atrophy since atrophy would affect all frames of the late binding phase at a similar magnitude. Future studies should investigate the impact of atrophy on kinetic modeling parameters by comparison of patients with mild and strong neurodegeneration. Future work should also investigate the impact of factors associated with tau binding (such as *APOE*) on kinetic modeling parameters in larger cohorts. We note that diagnoses of the heterogeneous patient cohort were built on clinical diagnosis criteria, but they were lacking postmortem validation. Therefore, clinical misdiagnosis could bias the primary outcome measures of this study. Because clinical 3R tauopathy patients were not enrolled in this study, we were not able to investigate the kinetic and binding characteristics of [^18^F]PI-2620 to 3R tau *in vivo.*

In conclusion, [^18^F]PI-2620 binding characteristics in cortical regions differentiate between 3/4R- and 4R-tauopathies and might serve as a supportive read-out in the diagnostic workup of neurodegenerative disorders. Higher tracer clearance in 4R tauopathies indicates less stable binding when compared to 3/4R-tauopathies, deserving attention when performing comparative studies between patients with presence of different tau isoforms.

## Supplemental Material

sj-pdf-1-jcb-10.1177_0271678X211018904 - Supplemental material for Binding characteristics of [^18^F]PI-2620 distinguish the clinically predicted tau isoform in different tauopathies by PET Click here for additional data file. Supplemental material, sj-pdf-1-jcb-10.1177_0271678X211018904 for Binding characteristics of [^18^F]PI-2620 distinguish the clinically predicted tau isoform in different tauopathies by PET by Mengmeng Song, Leonie Beyer, Lena Kaiser, Henryk Barthel, Thilo van Eimeren, Ken Marek, Alexander Nitschmann, Maximilian Scheifele, Carla Palleis, Gesine Respondek, Maike Kern, Gloria Biechele, Jochen Hammes, Gèrard Bischof, Michael Barbe, Özgür Onur, Frank Jessen, Dorothee Saur, Matthias L Schroeter, Jost-Julian Rumpf, Michael Rullmann, Andreas Schildan, Marianne Patt, Bernd Neumaier, Olivier Barret, Jennifer Madonia, David S Russell, Andrew W Stephens, Andre Mueller, Sigrun Roeber, Jochen Herms, Kai Bötzel, Adrian Danek, Johannes Levin, Joseph Classen, Günter U Höglinger, Peter Bartenstein, Victor Villemagne, Alexander Drzezga, John Seibyl, Osama Sabri, Guido Boening, Sibylle Ziegler and Matthias Brendel in Journal of Cerebral Blood Flow & Metabolism
