# Supplementary material for: Binding characteristics of [18F]PI-2620 distinguish the clinically predicted tau isoform in different tauopathies by PET
Source: J Cereb Blood Flow Metab. 2021 May 27;41(11):2957–72. doi: 10.1177/0271678X211018904 (PMC8545042; doi:10.1177/0271678X211018904)
Supplement: sj-pdf-1-jcb-10.1177_0271678X211018904 - Supplemental material for Binding characteristics of [18F]PI-2620 distinguish the clinically predicted tau isoform in different tauopathies by PET [file sj-pdf-1-jcb-10.1177_0271678X211018904.pdf]

Supplemental Figure 1

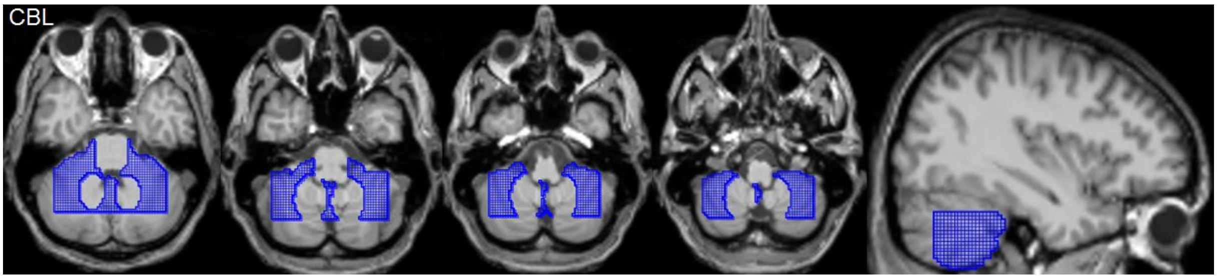

Supplemental Figure 1. Definition of the cerebellar reference tissue [1]. Definition of the cerebellar reference region (blue) was performed in the Montreal Neurology Institute (MNI) space. The outlined volume-of-interest is projected upon an MRI template, defined by the Hammers atlas[5]. Superior and posterior layers (1.5 cm) of the cerebellum were excluded manually in the MNI space. The dentate nucleus and the central cerebellar white matter were also excluded. CBL = cerebellum

Supplemental Figure 2

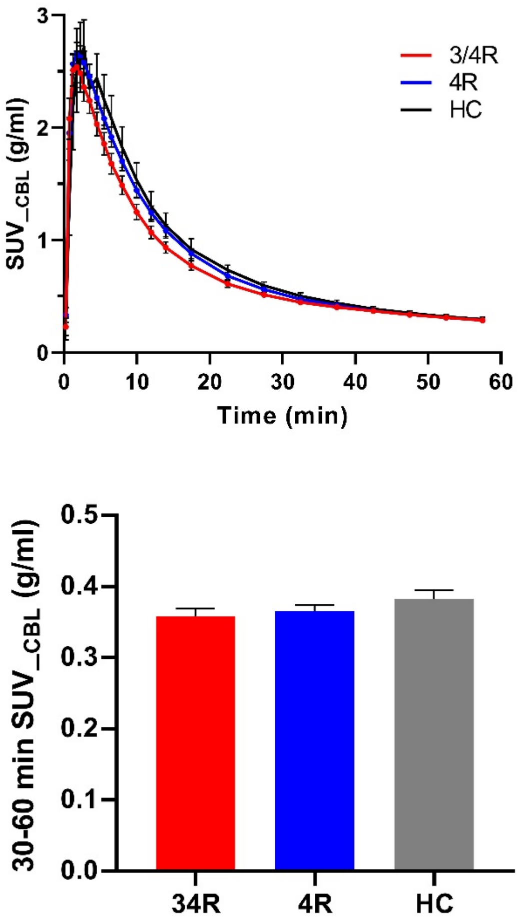

Supplemental Figure 2. Time-SUV curves and 30-60 min standardized uptake value (SUV) of the cerebellar reference tissue in comparison of 3/4R tauopathies (3/4R), 4R tauopathies (4R) and healthy controls (HC). Error bars represent SEM.

Supplemental Figure 3

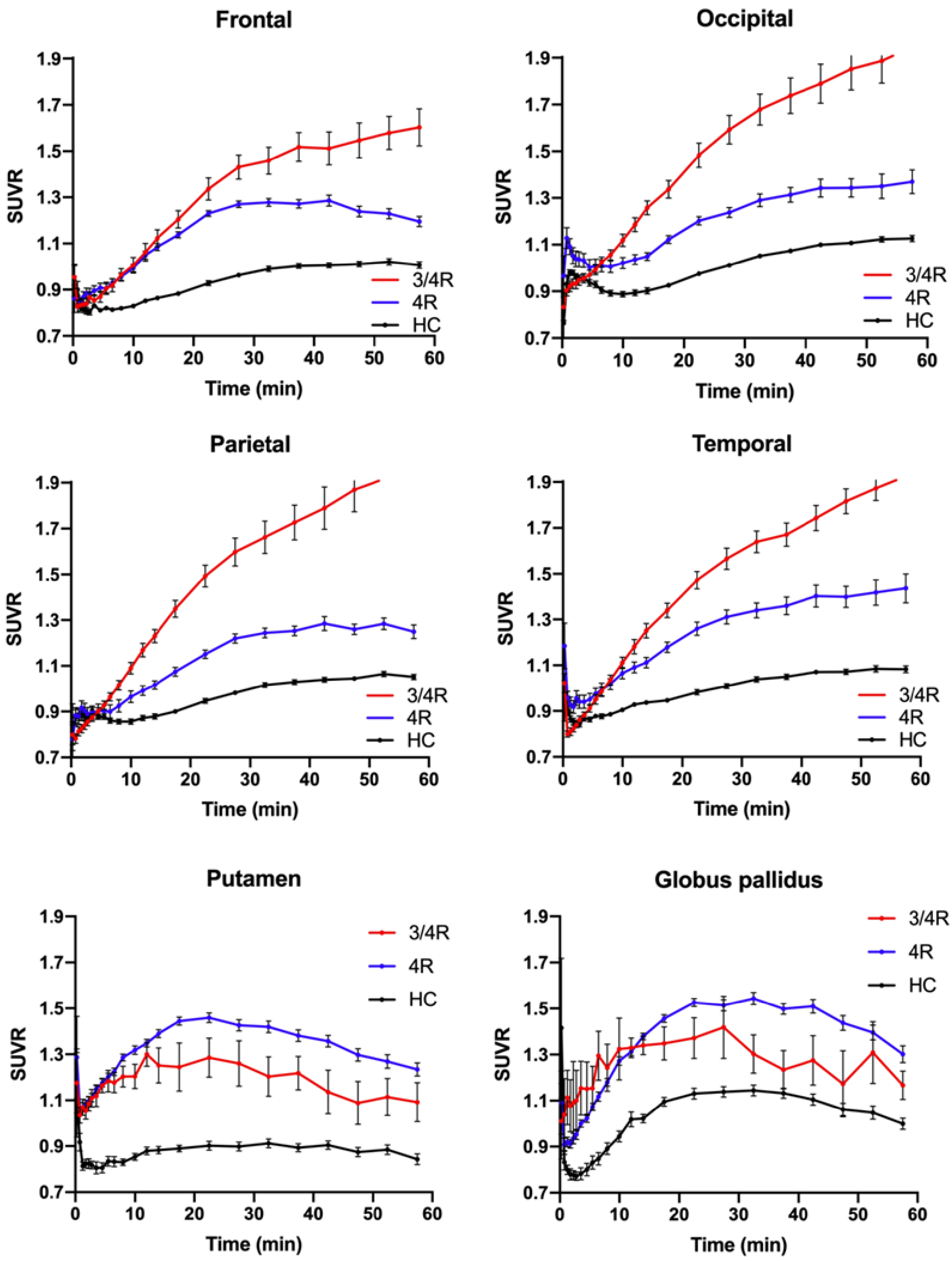

Supplemental Figure 3. Time–SUVR curves in the comparison of clinically diagnosed patients with 3/4R or 4R tauopathies and healthy controls for different cortical and subcortical subregions. Values of healthy controls (HC) were extracted from atlas regions, whereas values of 3/4R and 4R tauopathies were derived from [^{18}F]PI-2620 positive clusters.

Supplemental references:

1. Brendel M, Barthel H, van Eimeren T, Marek K, Beyer L, Song M *et al.* Assessment of 18F-PI-2620 as a Biomarker in Progressive Supranuclear Palsy. *JAMA Neurol* 2020; 77(11)**:** 1408-1419.

2. Keuken MC, Bazin PL, Backhouse K, Beekhuizen S, Himmer L, Kandola A *et al.* Effects of aging on T(1), T(2)*, and QSM MRI values in the subcortex. *Brain Struct Funct* 2017; 222(6)**:** 2487-2505.

3. Fan L, Li H, Zhuo J, Zhang Y, Wang J, Chen L *et al.* The Human Brainnetome Atlas: A New Brain Atlas Based on Connectional Architecture. *Cereb Cortex* 2016; 26(8)**:** 3508-26.

4. Carlen M. What constitutes the prefrontal cortex? *Science* 2017; 358(6362)**:** 478-482.

5. Hammers A, Allom R, Koepp MJ, Free SL, Myers R, Lemieux L *et al.* Three-dimensional maximum probability atlas of the human brain, with particular reference to the temporal lobe. *Hum Brain Mapp* 2003; 19(4)**:** 224-47.
